# Supplementary material for: Evaluation of Sustainable Recycled Products to Increase the Production of Nutraceutical and Antibacterial Molecules in Basil Plants by a Combined Metabolomic Approach
Source: Plants (Basel). 2023 Jan 23;12(3):513. doi: 10.3390/plants12030513 (PMC9919386; doi:10.3390/plants12030513)

Evaluation of sustainable recycled products to increase the production of nutraceutical molecules in basil plants by combined metabolomic approach

Mariavittoria Verrillo[1,2], Gunda Koellensperger[3,4], Marlene Puehringer[3,4], Alessandro Piccolo[1,2], Vincenza Cozzolino[1,2], Riccardo Spaccini[1,2Ɣ*], Evelyn Rampler [3,4Ɣ*]

1. Dipartimento di Agraria, Università di Napoli Federico II, Via Università 100, 80055 Portici, Italy
2. Centro Interdipartimentale di Ricerca per la Risonanza Magnetica Nucleare per l'Ambiente, l'Agroalimentare, ed i Nuovi Materiali (CERMANU), Università di Napoli Federico II, Via Università 100, 80055 Portici, Italy
3. Department of Analytical Chemistry, Faculty of Chemistry, University of Vienna, Waehringer Strasse 38, Vienna, 1090, Austria
4. Vienna Metabolomics Center (VIME), University of Vienna, Althanstrasse 14, Vienna, 1090, Austria

*Corresponding authors: Evelyn Rampler, phone: +43-1-4277-52381, evelyn.rampler@univie.ac.at; ORCID: https://orcid.org/0000-0002-9429-7663; Riccardo Spaccini: phone +39 0812539176, riccardo.spaccini@unina.it, ORCID https://orcid.org/0000-0002-9828-1992

Ɣ These authors contributed equally to this work

Table S1: Composition of multi-metabolite standard for metabolomics analysis.

	Compound Name	m/z	Adduct	Charge
1	1-Methylhydantoin	113.03565	M-H	-1
2	1-Methylnicotinamide	135.05639	M-H	-1
3	2'-Deoxyuridine	227.06734	M-H	-1
4	2-(Carbamoylamino)butanedioic acid	175.03604	M-H	-1
5	2-Deoxycytidine	226.08333	M-H	-1
6	3-Methyl-2-oxovaleric acid	129.05572	M-H	-1
7	3-Methylcytidine	256.09389	M-H	-1
8	3-Phosphoglycerate	184.98566	M-H	-1
9	3AMP	346.05581	M-H	-1
10	4-Hydroxy-proline	130.05097	M-H	-1
11	5'-Deoxy-5'-Methylthioadenosine	296.08228	M-H	-1
12	5-Methyluridine	257.07791	M-H	-1
13	6-Phosphogluconate	275.01736	M-H	-1
14	Adenine	134.04722	M-H	-1
15	Adenosine	266.08948	M-H	-1
16	ADP	426.02214	M-H	-1
17	Alanine	88.0404	M-H	-1
18	alpha-Aminoadipic acid	160.06153	M-H	-1
19	alpha-Ketoglutarate	145.01425	M-H	-1
20	AMP	346.05581	M-H	-1
21	Arginine	173.1044	M-H	-1
22	Argininosuccinic acid	289.11536	M-H	-1
23	Asparagine	131.04622	M-H	-1
24	Aspartate	132.03023	M-H	-1
25	ATP	505.98847	M-H	-1
26	Betaine	116.0717	M-H	-1
27	Biotin	243.08089	M-H	-1
28	cAMP	328.04524	M-H	-1
29	Carnitine	160.09792	M-H	-1
30	cGMP	344.04016	M-H	-1
31	Choline chloride	102.09244	M-H	-1
32	cis-Aconitate	173.00916	M-H	-1
33	Citrate	191.01973	M-H	-1
34	Citrulline	174.08841	M-H	-1
35	CMP	322.04457	M-H	-1
36	CTP	481.97723	M-H	-1
37	Cystathionine	221.06015	M-H	-1
38	Cysteic acid	167.99722	M-H	-1

39	Cysteine	120.01247	M-H	-1
40	Cysteinyl-glycine	177.03394	M-H	-1
41	Cystine	239.01657	M-H	-1
42	Cytidine	242.07824	M-H	-1
43	Cytosine	110.03599	M-H	-1
44	dAMP	330.06089	M-H	-1
45	dATP	489.99355	M-H	-1
46	dCMP	306.04966	M-H	-1
47	dCTP	465.98232	M-H	-1
48	dGTP	505.98847	M-H	-1
49	Dihydroxyacetonephosphate	168.99075	M-H	-1
50	Dihydroxyisovalerate	133.05063	M-H	-1
51	Erythrol	121.05063	M-H	-1
52	Erythrose-4-phosphate	199.00131	M-H	-1
53	Flavinadenin dinucleotide	784.14986	M-H	-1
54	Fructose	179.05611	M-H	-1
55	Fructose-1,6-bisphosphate	338.98877	M-H	-1
56	Fructose-6-phosphate	259.02244	M-H	-1
57	Fumarate	115.00368	M-H	-1
58	Galactose	179.05611	M-H	-1
59	GDP	442.01705	M-H	-1
60	Gluconate	195.05103	M-H	-1
61	Glucose	179.05611	M-H	-1
62	Glucose-1-phosphate	259.02244	M-H	-1
63	Glucose-6-phosphate	259.02244	M-H	-1
64	Glutamate	146.04588	M-H	-1
65	Glutamine	145.06187	M-H	-1
66	Glutamyl-cysteine	249.05507	M-H	-1
67	Glutathione, oxidized	611.14468	M-H	-1
68	Glutathione, reduced	306.07653	M-H	-1
69	Glycine	74.02475	M-H	-1
70	Glyoxylic acid	72.99312	M-H	-1
71	GMP	362.05072	M-H	-1
72	GTP	521.98338	M-H	-1
73	Guanidineacetic acid	116.04655	M-H	-1
74	Guanine	150.04213	M-H	-1
75	Guanosine	282.08439	M-H	-1
76	Histidine	154.0622	M-H	-1
77	Homocysteine	134.02812	M-H	-1
78	Homoserine	118.05097	M-H	-1
79	Hydroxyglutaric acid	147.0299	M-H	-1
80	IMP	347.03982	M-H	-1
81	Inosine	267.07349	M-H	-1
82	Inositol	179.05611	M-H	-1
83	Isocitrate	191.01973	M-H	-1
84	Isoguanosine	282.08439	M-H	-1

85	Isoleucine	130.08735	M-H	-1
86	Ketoisovalerate	115.04007	M-H	-1
87	Kynurenine	207.07752	M-H	-1
88	Lactate	89.02442	M-H	-1
89	Leucine	130.08735	M-H	-1
90	Lysine	145.09825	M-H	-1
91	Malate	133.01425	M-H	-1
92	Mannitol	181.07176	M-H	-1
93	Mannitol 1-phosphate	261.03809	M-H	-1
94	Mannose	179.05611	M-H	-1
95	Melatonine	231.1139	M-H	-1
96	Methionine	148.04377	M-H	-1
97	Methionine sulfone	180.0336	M-H	-1
98	Mevalonic acid	147.06628	M-H	-1
99	N-Acetyl-Asp-Glu	303.08339	M-H	-1
100	N-Acetyl-L-aspartic acid	174.0408	M-H	-1
101	N-Acetyl-serine	146.04588	M-H	-1
102	N4-Acetylcytidine	284.08881	M-H	-1
103	NAD+	662.10184	M-H	-1
104	NADH	664.11749	M-H	-1
105	NADP+	742.06817	M-H	-1
106	NADPH	744.08382	M-H	-1
107	Nicotinamide	121.04074	M-H	-1
108	Octopamine	152.0717	M-H	-1
109	Ornithine	131.0826	M-H	-1
110	Oxaloacetic acid	130.9986	M-H	-1
111	Palmitic acid	255.23295	M-H	-1
112	Phenylalanine	164.0717	M-H	-1
113	Phosphocreatine	210.02853	M-H	-1
114	Proline	114.05605	M-H	-1
115	Propionyl-L-carnitine	216.12413	M-H	-1
116	Pseudouridine	243.06226	M-H	-1
117	Pyruvate	87.00877	M-H	-1
118	Ribose	149.04555	M-H	-1
119	Ribose-5-phosphate	229.01188	M-H	-1
120	Ribulose-5-phosphate	229.01188	M-H	-1
121	S-(Adenosyl)-methionine	397.12996	M-H	-1
122	S-Adenosyl-homocysteine	383.11431	M-H	-1
123	Sarcosine	88.0404	M-H	-1
124	Sedoheptulose-7-phosphate	289.03301	M-H	-1
125	Seleno-methionine	195.98822	M-H	-1
126	Serine	104.03532	M-H	-1
127	Serotonine	175.08769	M-H	-1
128	Spermidine	144.15062	M-H	-1
129	Spermine	201.20847	M-H	-1
130	Succinate	117.01933	M-H	-1

131	Thiamine	264.10503	M-H	-1
132	Threonine	118.05097	M-H	-1
133	Thymidine	241.08299	M-H	-1
134	Thymine	125.03565	M-H	-1
135	TMP	321.04933	M-H	-1
136	Trehalose	341.10893	M-H	-1
137	Tryptophan	203.0826	M-H	-1
138	TTP	480.98199	M-H	-1
139	Tyrosine	180.06662	M-H	-1
140	Methylhydantoin	117.04907	M-H	-1
141	Methylnicotinamide	142.07987	M-H	-1
142	Deoxyuridine	236.09754	M-H	-1
143	(Carbamoylamino)butanedioic acid	180.05282	M-H	-1
144	Deoxycytidine	235.11352	M-H	-1
145	3-Methyl-2-oxovaleric acid	135.07585	M-H	-1
146	Methylcytidine	266.12744	M-H	-1
147	3-Phosphoglycerate	187.99573	M-H	-1
148	AMP	356.08936	M-H	-1
149	4-Hydroxy-proline	135.06774	M-H	-1
150	5'-Deoxy-5'-Methylthioadenosine	307.11919	M-H	-1
151	Methyluridine	267.11146	M-H	-1
152	Phosphogluconate	281.03749	M-H	-1
153	Adenine	139.06399	M-H	-1
154	Adenosine	276.12303	M-H	-1
155	ADP	436.05569	M-H	-1
156	Alanine	91.05047	M-H	-1
157	alpha-Aminoadipic acid	166.08166	M-H	-1
158	alpha-Ketoglutarate	150.03102	M-H	-1
159	Arginine	179.12453	M-H	-1
160	Argininosuccinic acid	299.14891	M-H	-1
161	Asparagine	135.05963	M-H	-1
162	Aspartate	136.04365	M-H	-1
163	ATP	516.02202	M-H	-1
164	Betaine	121.08848	M-H	-1
165	Biotin	253.11443	M-H	-1
166	cAMP	338.07879	M-H	-1
167	Carnitine	167.1214	M-H	-1
168	cGMP	354.07371	M-H	-1
169	Choline chloride	107.10921	M-H	-1
170	cis-Aconitate	179.02929	M-H	-1
171	Citrate	197.03985	M-H	-1
172	Citrulline	180.10854	M-H	-1
173	CMP	331.07477	M-H	-1
174	CTP	491.00743	M-H	-1
175	Cystathionine	228.08363	M-H	-1
176	Cysteic acid	171.00728	M-H	-1

177	Cysteine	123.02254	M-H	-1
178	Cysteinyl-glycine	182.05071	M-H	-1
179	Cystine	245.0367	M-H	-1
180	Cytidine	251.10844	M-H	-1
181	Cytosine	114.0494	M-H	-1
182	dAMP	340.09444	M-H	-1
183	dATP	500.0271	M-H	-1
184	dCMP	315.07985	M-H	-1
185	dCTP	475.01251	M-H	-1
186	dGTP	516.02202	M-H	-1
187	Dihydroxyacetonephosphate	172.00081	M-H	-1
188	Dihydroxyisovalerate	138.06741	M-H	-1
189	Erythrol	125.06405	M-H	-1
190	Erythrose-4-phosphate	203.01473	M-H	-1
191	Flavinadenin dinucleotide	811.24044	M-H	-1
192	Fructose	185.07624	M-H	-1
193	Fructose-1,6-bisphosphate	345.0089	M-H	-1
194	Fructose-6-phosphate	265.04257	M-H	-1
195	Fumarate	119.0171	M-H	-1
196	Galactose	185.07624	M-H	-1
197	GDP	452.0506	M-H	-1
198	Gluconate	201.07115	M-H	-1
199	Glucose	185.07624	M-H	-1
200	Glucose-1-phosphate	265.04257	M-H	-1
201	Glucose-6-phosphate	265.04257	M-H	-1
202	Glutamate	151.06266	M-H	-1
203	Glutamine	150.07864	M-H	-1
204	Glutamyl-cysteine	257.0819	M-H	-1
205	Glutathione, oxidized	631.21178	M-H	-1
206	Glutathione, reduced	316.11008	M-H	-1
207	Glycine	76.03146	M-H	-1
208	Glyoxylic acid	74.99983	M-H	-1
209	GMP	372.08427	M-H	-1
210	GTP	532.01693	M-H	-1
211	Guanidineacetic acid	119.05661	M-H	-1
212	Guanine	155.05891	M-H	-1
213	Guanosine	292.11794	M-H	-1
214	Histidine	160.08233	M-H	-1
215	Homocysteine	138.04154	M-H	-1
216	Homoserine	122.06439	M-H	-1
217	Hydroxyglutaric acid	152.04667	M-H	-1
218	IMP	357.07337	M-H	-1
219	Inosine	277.10704	M-H	-1
220	Inositol	185.07624	M-H	-1
221	Isocitrate	197.03985	M-H	-1
222	Isoguanosine	292.11794	M-H	-1

223	Isoleucine	136.10748	M-H	-1
224	Ketoisovalerate	120.05684	M-H	-1
225	Kynurenine	217.11106	M-H	-1
226	Lactate	92.03448	M-H	-1
227	Leucine	136.10748	M-H	-1
228	Lysine	151.11838	M-H	-1
229	Malate	137.02767	M-H	-1
230	Mannitol	187.09189	M-H	-1
231	Mannitol 1-phosphate	267.05822	M-H	-1
232	Mannose	185.07624	M-H	-1
233	Melatonine	244.15751	M-H	-1
234	Methionine	153.06055	M-H	-1
235	Methionine sulfone	185.05038	M-H	-1
236	Mevalonic acid	153.08641	M-H	-1
237	N-Acetyl-Asp-Glu	314.12029	M-H	-1
238	N-Acetyl-L-aspartic acid	180.06092	M-H	-1
239	N-Acetyl-serine	151.06266	M-H	-1
240	N4-Acetylcytidine	295.12571	M-H	-1
241	NAD+	683.1723	M-H	-1
242	NADH	685.18795	M-H	-1
243	NADP+	763.13863	M-H	-1
244	NADPH	765.15428	M-H	-1
245	Nicotinamide	127.06086	M-H	-1
246	Octopamine	160.09854	M-H	-1
247	Ornithine	136.09937	M-H	-1
248	Oxaloacetic acid	135.01202	M-H	-1
249	Palmitic acid	271.28663	M-H	-1
250	Phenylalanine	173.1019	M-H	-1
251	2-phosphoenolpyruvate, PEP	167.042	M-H	-1
252	Phosphocreatine	214.04195	M-H	-1
253	Proline	119.07283	M-H	-1
254	Propionyl-L-carnitine	226.15768	M-H	-1
255	Pseudouridine	252.09245	M-H	-1
256	Pyruvate	90.01883	M-H	-1
257	Ribose	154.06232	M-H	-1
258	Ribose-5-phosphate	234.02865	M-H	-1
259	Ribulose-5-phosphate	234.02865	M-H	-1
260	S-(Adenosyl)-methionine	412.18028	M-H	-1
261	S-Adenosyl-homocysteine	397.16128	M-H	-1
262	Sarcosine	91.05047	M-H	-1
263	Sedoheptulose-7-phosphate	296.05649	M-H	-1
264	Seleno-methionine	201.005	M-H	-1
265	Serine	107.04538	M-H	-1
266	Serotonine	185.12123	M-H	-1
267	Spermidine	151.1741	M-H	-1
268	Spermine	211.24202	M-H	-1

269	Succinate	121.03275	M-H	-1
270	Thiamine	276.14529	M-H	-1
271	Threonine	122.06439	M-H	-1
272	Thymidine	251.11654	M-H	-1
273	Thymine	130.05242	M-H	-1
274	TMP	331.08287	M-H	-1
275	Trehalose	353.14919	M-H	-1
276	Tryptophan	214.1195	M-H	-1
277	TTP	491.01553	M-H	-1
278	Tyrosine	189.09681	M-H	-1
279	UDP	412.02511	M-H	-1
280	UMP	332.05878	M-H	-1
281	Uracil	115.03342	M-H	-1
282	Urea	60.02844	M-H	-1
283	Uridine	252.09245	M-H	-1
284	UTP	491.99144	M-H	-1
285	Valine	121.08848	M-H	-1
286	Xanthine	156.04292	M-H	-1
287	Xylose	154.06232	M-H	-1
288	UDP	402.99492	M-H	-1
289	UMP	323.02859	M-H	-1
290	Uracil	111.02	M-H	-1
291	Urea	59.02509	M-H	-1
292	Uridine	243.06226	M-H	-1
293	UTP	482.96125	M-H	-1
294	Valine	116.0717	M-H	-1
295	Xanthine	151.02615	M-H	-1
296	Xylose	149.04555	M-H	-1
297	Rosmarinic Acid	359.0845	M-H	-1
298	Caffeic acid	179.042252	M-H	-1
299	Epicatechin	289.079041	M-H	-1
300	Kaempferol	285.047729	M-H	-1
301	Salvianolic acid B	717.153381	M-H	-1
302	Trans Cinnamic Acid	149.052429	M-H	-1
303	Resveratrol	227.078644	M-H	-1
304	p-Coumaric acid	163.047348	M-H	-1
305	Tocopherol	429.381073	M-H	-1
306	4-Hydroxy benzoic acid	137.031693	M-H	-1
307	Chicoric Acid	473.079834	M-H	-1
308	Quercetin-glucoside	319.053223	M-H	-1
309	Naringenine	271.25	M-H	-1

Table S2: Identification of primary and secondary metabolites from basil leaves by Liquid Chromatography High-Resolution Mass Spectrometry (LC-HRMS).

	Metabolite	Molecular Formula	M.W.[a]	R.T.[a]
1	Arginine	C6 H14 N4 O2	174.11145	1.599
2	α-Trehalose	C12 H22 O11	342.11597	1.621
4	Serine	C3 H7 N O3	105.04243	1.626
5	Arginine	C6 H14 N4 O2	174.11148	1.633
6	5,5-Dimethylhydantoin	C5 H8 N2 O2	128.05845	1.666
7	Glutamine	C5 H10 N2 O3	146.06899	1.666
8	Threonine	C4 H9 N O3	119.05804	1.668
9	Aspartic acid	C4 H7 N O4	133.03737	1.669
11	Glutamic acid	C5 H9 N O4	147.053	1.691
12	Glucose	C6 H12 O6	226.06844	1.716
13	α-Lactose	C12 H22 O11	388.12125	1.729
14	Mannose	C6 H12 O6	180.06319	1.732
15	Gluconic acid	C6 H12 O7	150.05233	1.74
16	Threonic acid	C4 H8 O5	136.03706	1.796
17	7-Methylxanthine	C6 H6 N4 O2	166.0475	1.824
18	N-Acetylornithine	C7 H14 N2 O3	174.10019	1.827
19	Quinic acid	C7 H12 O6	192.06329	1.832
20	2,3-Dihydroxypropanoic acid	C3 H6 O4	106.0265	1.883
21	δ-Gluconic acid δ-lactone	C6 H10 O6	132.04214	1.942
22	2,4-Dinitrophenol	C6 H4 N2 O5	184.01361	1.998
23	Purine	C5 H4 N4	120.04204	2.005
24	Quinic acid	C7 H12 O6	192.06313	2.008
25	Isocitric acid	C6 H8 O7	146.02134	2.019

26	Malic acid	C4 H6 O5	134.02135	2.036
27	Fructose	C6 H12 O6	180.06319	2.085
28	Tartaric acid	C4 H6 O6	150.01622	2.092
29	Fumaric acid	C4 H4 O4	116.01082	2.177
30	Uridine monophosphate (UMP)	C9 H13 N2 O9 P	324.0353	2.179
31	Adenosine 5'-monophosphate	C10 H14 N5 O7 P	347.06244	2.211
32	2-Oxobutyric acid	C4 H6 O3	102.03161	2.253
33	Citric acid	C6 H8 O7	192.02682	2.413
34	Pseudouridine	C9 H12 N2 O6	244.06903	2.421
35	2-Oxoglutaric acid	C5 H6 O5	146.0213	2.585
36	2-Furoic acid	C5 H4 O3	112.01592	2.608
37	Mesaconic acid	C5 H6 O4	130.0264	2.609
38	Uridine	C9 H12 N2 O6	244.06928	2.624
39	4-Oxoproline	C5 H7 N O3	129.04251	2.64
40	Adenosine	C10 H13 N5 O4	267.09655	2.668
41	2-Aminoadipic acid	C6 H11 N O4	161.06869	2.675
42	Tyrosine	C9 H11 N O3	181.07375	2.728
43	N-Acetyl-DL-glutamic acid	C7 H11 N O5	189.06355	2.761
44	Endothal	C8 H10 O5	186.05261	2.773
45	3-Hydroxybutyric acid	C4 H8 O3	104.04733	2.801
46	Guanosine	C10 H13 N5 O5	283.09138	2.838
47	Citraconic acid	C5 H6 O4	130.02639	2.849
48	cis-Aconitic acid	C6 H6 O6	174.01617	2.859
49	Leucine	C6 H13 N O2	131.09447	2.86
50	δ-Ribono-1,4-lactone	C5 H8 O5	148.03704	2.982
51	Succinic acid	C4 H6 O4	118.02647	3.038
52	4-Acetamidobutanoic acid	C6 H11 N O3	145.07376	3.523
53	N-Ethylglycine	C4 H9 N O2	103.06328	3.53
54	4-Pyridoxic acid	C8 H9 N O4	183.05306	3.551
55	Propanoic acid	C16 H22 O9	358.12629	3.605
56	Catechol	C6 H6 O2	110.03665	3.755
57	Xanthosine	C10 H12 N4 O6	284.07547	3.769

58	Gallic acid	C7 H6 O5	170.02144	4.183
59	Itaconic acid	C5 H6 O4	130.02649	4.366
60	N-Acetyl-4-aminosalicylic acid	C9 H9 N O4	195.05298	4.45
61	2-Aminooctanedioic acid	C8 H15 N O4	189.10001	4.497
62	Glutaric acid	C5 H8 O4	132.04211	4.554
63	Galactose	C6 H12 O6	180.06323	4.625
64	Pantothenic acid	C9 H17 N O5	219.1105	4.639
65	Phenylalanine	C9 H11 N O2	165.07885	4.882
66	Pyrogallol	C6 H6 O3	126.03157	4.939
67	Porphobilinogen	C10 H14 N2 O4	226.09514	5.207
68	Sorbic acid	C6 H8 O2	112.05237	5.215
69	N-Acetylvaline	C7 H13 N O3	159.08938	5.219
70	3-Anisic acid	C8 H8 O3	152.04714	5.257
71	Gentisic acid	C7 H6 O4	154.0264	5.258
72	Methylsuccinic acid	C5 H8 O4	132.04215	5.399
73	Ethylmalonic acid	C5 H8 O4	132.04211	5.4
74	2-Methylbenzoic acid	C8 H8 O2	136.05233	5.424
75	4-Hydroxyphenylacetic acid	C8 H8 O3	152.04717	5.424
76	6-Methoxysalicylic acid	C8 H8 O4	168.04214	5.514
77	Methyl salicylate	C8 H8 O3	152.04713	5.525
78	4-Hydroxybenzylalcohol	C7 H8 O2	124.05237	5.536
79	Xanthurenic acid	C10 H7 N O4	205.03731	5.614
80	Benzoic acid	C7 H6 O2	122.03664	5.687
81	Adenine	C5 H5 N5	135.0544	5.825
82	5'-S-Methyl-5'-thioadenosine	C11 H15 N5 O3 S	297.08929	5.826
83	Adipic acid	C6 H10 O4	146.05777	6.024
84	Tryptophan	C11 H12 N2 O2	204.08977	6.081
85	2,4-Dihydroxybenzoic acid	C7 H6 O4	154.02647	6.095
86	Caffeic acid	C9 H8 O4	134.03656	6.182
87	Syringic acid	C9 H10 O5	198.0526	6.245
88	Esculin	C15 H16 O9	340.07913	6.274
89	Geniposide	C17 H24 O10	388.13684	6.321

90	4-Methylumbelliferone	C10 H8 O3	176.0471	6.357
91	4-Hydroxyphenyllactic acid	C9 H10 O4	182.0578	6.365
92	N-Acetyl-L-methionine	C7 H13 N O3 S	191.06143	6.367
93	Mesalamine	C7 H7 N O3	153.04228	6.603
94	Kynurenic acid	C10 H7 N O3	189.04248	6.651
95	Triethyl 4-phosphonocrotonate	C10 H19 O5 P	250.09683	6.716
96	2-Hydroxycinnamic acid	C9 H8 O3	164.04722	6.764
97	Salicylic acid	C7 H6 O3	138.03154	7.072
98	2,5-Dihydroxybenzaldehyde	C7 H6 O3	138.03137	7.207
99	Pimelic acid	C7 H12 O4	160.07339	7.285
100	Ferulic acid	C10 H10 O4	194.05771	7.37
101	Vanillic acid	C8 H8 O4	168.04208	7.454
102	Phenylglyoxylic acid	C8 H6 O3	150.03148	7.649
103	Astragalin	C21 H20 O11	448.10039	7.814
104	Rutin	C27 H30 O16	610.15302	8.027
105	Cyclopentylacetic acid	C7 H12 O2	128.0836	8.086
106	Lariciresinol 4-O-glucoside	C26 H34 O11	568.2154	8.249
107	Quercetin-3β-D-glucoside	C21 H20 O12	464.09515	8.392
108	Eriodictyol	C15 H12 O6	288.06325	8.406
109	N-Acetyl-L-phenylalanine	C11 H13 N O3	207.08929	8.496
110	Suberic acid	C8 H14 O4	174.08899	8.541
111	Dehydroacetic acid	C8 H8 O4	168.04205	8.908
112	Vanillin	C8 H8 O3	152.04714	8.989
113	Afzelin	C21 H20 O10	432.10545	9.085
114	Corey lactone	C8 H12 O4	172.07328	9.125
115	N-Acetyl-DL-tryptophan	C13 H14 N2 O3	246.10018	9.187
116	3-O-β-D-Glucopyranosylandrographolide	C26 H40 O10	558.26991	9.268
117	Dodecanedioic acid	C12 H22 O4	230.15149	9.359
118	6-Hydroxycaproic acid	C6 H12 O3	132.07847	9.512
119	Azelaic acid	C9 H16 O4	188.10465	9.738
120	3-Phenyllactic acid	C9 H10 O3	120.0573	9.975
121	trans-Cinnamic acid	C9 H8 O2	148.05227	9.975

122	4-Indolecarbaldehyde	C9 H7 N O	145.05259	10.029
123	Isoferulic acid	C10 H10 O4	194.05764	10.375
124	Eugenol	C10 H12 O2	164.08363	10.46
125	4-Acetamidobenzoic acid	C9 H9 N O3	179.05801	10.845
126	4-Nitrophenol	C6 H5 N O3	139.02676	10.931
127	Abscisic acid	C15 H20 O4	264.13598	10.974
128	2-Hydroxyphenylalanine	C9 H11 N O3	181.07379	11.088
129	Hydrocinnamic acid	C9 H10 O2	150.06786	11.657
130	Genipin	C11 H14 O5	208.07338	11.661
131	Genistein	C15 H10 O5	270.0526	12.017
132	Naringenin	C15 H12 O5	272.0682	12.024
133	Formononetin	C16 H12 O4	268.07336	12.054
134	Hispidulin	C16 H12 O6	300.06317	12.215
135	3',5,7-Trihydroxy-4'-methoxyflavanone	C16 H14 O6	302.07883	12.231
136	Trihydroxy-octadecenoic acid	C18 H34 O5	330.24048	12.329
137	Helvolic acid	C33 H44 O8	568.30693	12.35
138	Jasmonic acid	C12 H18 O3	210.12539	12.354
139	4-Hydroxycoumarin	C9 H6 O3	162.03157	12.37
140	3-Hydroxyanthranilic acid	C7 H7 N O3	153.04245	12.568
141	Arjungenin	C30 H48 O6	504.34491	12.883
142	Hydroxydodecanoic acid	C12 H24 O3	216.172	13.449
143	Hexadecanedioic acid	C16 H30 O4	286.21388	13.851
144	Butylparaben	C11 H14 O3	194.0941	13.962
145	2,5-di-tert-Butylhydroquinone	C14 H22 O2	222.16152	14.953
146	Asiatic acid	C30 H48 O5	488.34983	15.825
147	Hydroxyhexadecanoic acid	C16 H32 O3	272.23501	17.717
148	Abietic acid	C20 H30 O2	302.22436	18.161
149	Ostruthin	C19 H22 O3	298.16003	24.186

a MW= molecular weight; RT= Retention time (minutes)

Figure S1: Total Ion Chromatogram (TIC) by Liquid Chromatography High Resolution Mass Spectrometry (LC/HRMS) of basil leaves extract (CTRL sample).

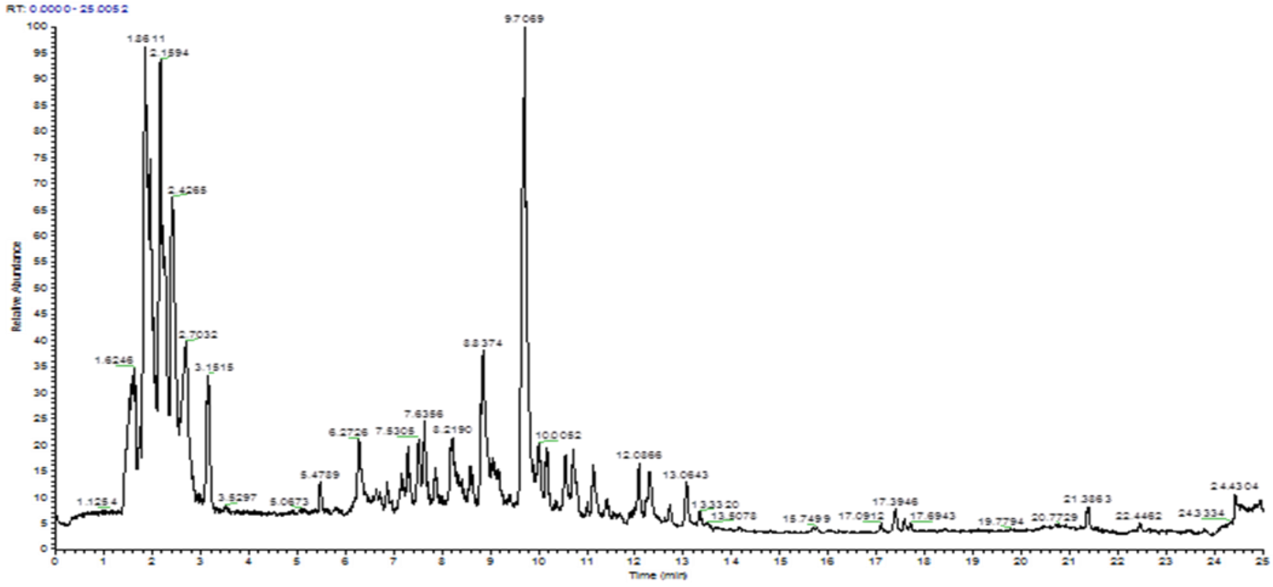

Supplement: Supplementary file 1 [file plants-12-00513-s001.zip › plants-2161514-supplementary.pdf]
